# Integrated comparative metabolomics and network pharmacology approach to uncover the key active ingredients of *Polygonati rhizoma* and their therapeutic potential for the treatment of Alzheimer’s disease

**DOI:** 10.3389/fphar.2022.934947

**Published:** 2022-08-04

**Authors:** Fu Wang, Hongping Chen, Yuan Hu, Lin Chen, Youping Liu

**Affiliations:** State Key Laboratory of Southwestern Chinese Medicine Resources, Department of Pharmacy, Chengdu University of TCM, Chengdu, Sichuan, China

**Keywords:** comparative metabolomics, network pharmacology, Alzheimer’s disease, key active ingredients, *Polygonati rhizoma*

## Abstract

Alzheimer’s disease (AD) has become a worldwide disease affecting human health and resulting in a heavy economic burden on the healthcare system. *Polygonati rhizoma* (PR), a kind of traditional Chinese medicine (TCM), is known to improve learning and memory abilities. However, its AD-treating material basis and therapeutic potential for the treatment of AD have remained unclear. Therefore, the present study aimed to uncover the key active ingredients of PR and its therapeutic potential for the treatment of AD. First, we used comparative metabolomics to identify the potential key active ingredients in the edible and medicinal PR. Second, network pharmacology was used to decipher the effects and potential targets of key active ingredients in the PR for the treatment of AD, and molecular docking was further used to identify the binding ability of those active ingredients with AD-related target of AChE. The rate of acetylcholinesterase (AChE) inhibition, oxidative stress, neuroprotective effects, and anti-inflammatory activity were assessed *in vitro* to screen the potential active ingredients in the PR with therapeutic potential against AD. Finally, APPswe/PS1dE9 AD mice were used to screen the therapeutic components in the PR. Seven overlapping upregulated differential metabolites were identified as the key active ingredients, among which cafestol, isorhamnetin, and rutin have AChE inhibitory activity, anti-inflammatory activity, and neuroprotective effects *in vitro* validation assays. Furthermore, *in vivo* results showed that cafestol, isorhamnetin, and rutin displayed several beneficial effects in AD transgenic mice by reducing the number of Aβ-positive spots and the levels of inflammatory cytokines, inhibiting the AChE activity, and increasing the antioxidant levels. Each compound is involved in a different function in the early stages of AD. In conclusion, our results corroborate the current understanding of the therapeutic effects of PR on AD. In addition, our work demonstrated that the proposed network pharmacology-integrated comparative metabolomics strategy is a powerful way of identifying key active ingredients and mechanisms contributing to the pharmacological effects of TCM.

## Introduction

Alzheimer’s disease (AD) is a neurodegenerative disease, primarily characterized by memory loss and cognitive decline, and accompanied by irreversible neuron loss, which occurs more often in the elderly over 65 years of age (Mantzavinos and Alexiou, 2017). More than 52 million patients with AD have been reported worldwide in 2020, and this number is estimated to increase to 152 million in 2050 (Patterson, 2018). Thus, AD has emerged as a serious global public health problem, which not only imposes a huge burden on the affected people and their families but also creates a huge economic pressure on society. Although the neuropathological features of AD have been recognized over the past several decades, the underlying pathological mechanisms have not been elucidated. Acetylcholinesterase (AChE), a key enzyme in biological nerve conduction, catalyzes the hydrolysis of acetylcholine (ACh), a neurotransmitter, to choline and acetic acid, thereby blocking the transmission of nerve signals (Thapa et al., 2017). Increased AChE activity can lead to decreased ACh levels, which is the key cause of AD. Therefore, a significant treatment strategy could be to inhibit the activity of AChE to improve the levels of ACh. To date, acetylcholinesterase inhibitors (AChEI) approved by the Food and Drug Administration (FDA) for the treatment of AD clinically majorly included taclin, donepezil, rivastigmine, and galantamine (Shaikh et al., 2014). Except for galantamine, the majority of them are synthetic drugs. Although they have certain therapeutic effects, they have different degrees of side effects such as dizziness, insomnia, nausea, and mild diarrhea, which are not suitable for long-term use by patients. Increasing evidence suggests that AD is a multifactorial disease; thus, the previous therapies targeting a single target are unsuitable. Hence, multi-component and multi-target natural products or herbal medicines could be the next choice for the treatment of AD.

Natural products and their derivatives have historically been invaluable as a source of therapeutic agents, the majority of new drugs have been generated from natural products. Natural products such as anthocyanin and vitamins and other compounds with unique features represent attractive sources to address complex diseases (Al-Shamsi et al., 2006a; Al-Shamsi et al., 2006b; Liu et al., 2021). Therefore, the research on natural products and their biological activities has attracted great attention in recent years. Meanwhile, natural products may become a successful and safe strategy to treat multifactorial diseases.

Traditional Chinese medicine (TCM) has a history of more than 3,000 years and is based on the concept of “multiple components, multiple targets, and multiple pathways.” There are several herbal medicines for treating AD (Pei et al., 2020). *Polygonati rhizoma* (PR, Chinese name: Huangjing), a kind of herbal medicine, has properties of lowering blood pressure, lowering blood sugar, improving coronary artery, anti-inflammation, antivirus, immune regulation, anti-oxidation, and scavenging free radicals, with great value for the treatment of AD (Wang et al., 2019). In addition, it has been reported that PR can improve the learning and memory ability of AD rats. However, its AD-treating material basis and therapeutic potential for the treatment of AD have remained elusive.

Network pharmacology combines systems biology, computational biology, and other multidisciplinary technologies to construct a complex network between metabolites, their prospective targets, and different diseases, enabling us to identify the relevant pathways and clarify the therapeutic mechanisms (Wang et al., 2021). Accumulating evidence has demonstrated that it has great advantages in discovering multi-target drugs and providing a new insight into studying TCM (Zhou Z. et al., 2020). Comparative metabolomics was identified as an emerging system biology technology that compares the metabolites of the control and experimental groups to identify the differences in their metabolic profiles (Rashid et al., 2021). Furthermore, the systematic biological characteristics of comparative metabolomics are consistent with the theory of TCM, allowing us to thoroughly investigate TCM with complicated conditions and multiple factors (Qu et al., 2021). Therefore, a study combining network pharmacology with comparative metabolomics may be performed to comprehensively elaborate on the underlying mechanism of AD.

**FIGURE 1:**
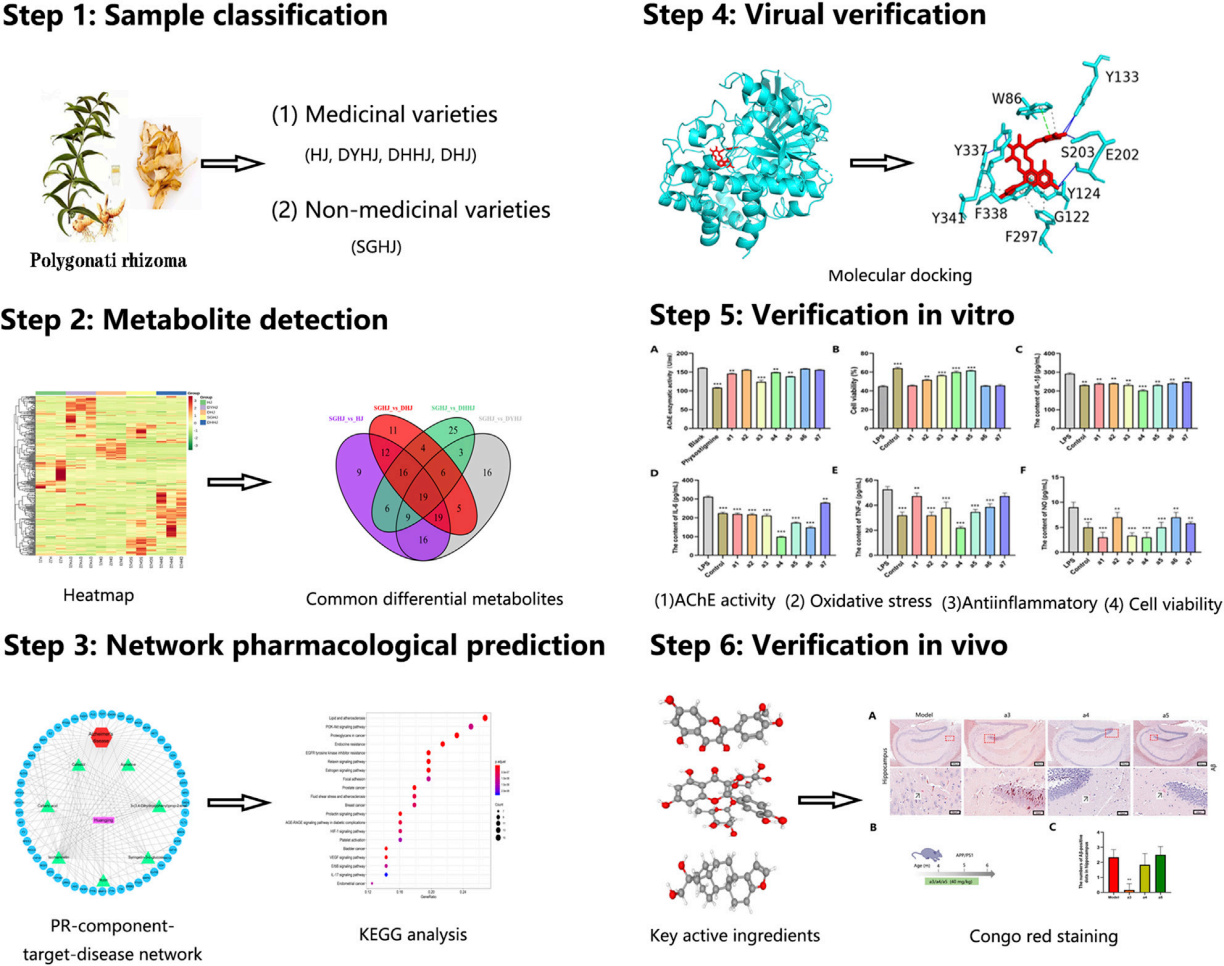
The integrated strategy of comparative metabolomics and network pharmacology.

In our study, comparative metabolomics was first used to identify the potential key active ingredients. Next, network pharmacology was applied to predict the key active ingredient targets of PR for treating AD. Finally, several key active ingredients from PR were used *in vitro* and *in vivo* experimental validation to assess their actual effects on the treatment of AD. To the best of our knowledge, this is the first study to investigate the therapeutic mechanisms of PR on AD using a combination of comparative metabolomics and a network pharmacology approach (Figure 1).

## Materials and methods

### Materials

Agmatine, caftaric acid, isorhamnetin, rutin, cafestol, syringetin-3-O-glucoside, and 3-(3,4-dihydroxyphenyl)acrylaldehyde were purchased from Chengdu Herbpurify Co., Ltd. (Chengdu, China). The assay kits for catalase (CAT), plasma glutathione peroxidase (GSH-Px), superoxide dismutase (SOD), and malondialdehyde (MDA) were obtained from Beyotime Co., Ltd. (Jiangsu, China). The interleukin (IL)-1 β, IL-6, tumor necrosis factor (TNF)-ɑ, and nitric oxide (NO) enzyme-linked immunosorbent assay (ELISA) kits were from R&D Systems (Minneapolis, MN, United States). CCK-8 cell proliferation and cytotoxicity test kits were from BIOSHARP (BS350B). The AChE kit was procured from Elabscience Biotechnology Co., Ltd. (Wuhan, China). Lipopolysaccharide (LPS) was from Zhang Jinlong Technology (Chengdu, China).

Samples used in this study were collected from Jialu Village, Xiushan Tujia Miao Autonomous County, Chongqing, China on 9 July 2020. All samples were identified by Yan Zhuyun, an expert in the Medicinal Resources Department of Chengdu University of TCM. The edible varieties (SGHJ) were classified as the control group and the medicinal varieties, namely, HJ, DHJ, DHHJ, and DYHJ, as the experimental group. SGHJ: *Polygonatum alternicirrhosum* Hand.-Mzt. HJ: *Polygonatum sibiricum* Red. DHJ: *Polygonatum kingianum* Coll.et Hemsl. DHHJ: *Polygonatum cyrtonema* Hua. DYHJ: *Polygonatum kingianum* var. grandifolium. SGHJ is a local wild edible variety, which is commonly known as a kind of fruit because of its high polysaccharide content and sweet taste. In addition, the other four varieties were both medicinal varieties. HJ, DHJ, and DHHJ are listed in the pharmacopoeia of the Peoples’ Republic of China (2020 edition). DYHJ is a TCM contained in local standards. Tubers of all the above samples were frozen in liquid nitrogen immediately after collection and stored at −80°C.

### Sample preparation

The samples were extracted and prepared using the method described by Li et al.(2022). Biological samples were freeze-dried by a vacuum freeze dryer (LGJ-8, Songyuan Technology, Beijing, China). Freeze-dried samples were crushed using a mixer mill (MM400; Retsch, Laichi, Germany) with a zirconia bead for 1.5 min at 30 Hz. Next, 100 mg of powder was dissolved with 1.2 ml 70% methanol solution, vortex 30 s every 30 min six times, and placed the sample in a refrigerator at 4°C overnight. Following centrifugation at 1,200 rpm for 10 min, the extracts were filtrated (SCAA-104, 0.22 μm pore size; ANPEL, Shanghai, China) before LC-MS analysis.

### UPLC conditions

The sample extracts were analyzed using a UPLC-ESI-MS/MS system (UPLC, SHIMADZU Nexera X2; MS, Applied Biosystems 4500 Q TRAP). The analytical conditions were UPLC: column, Agilent SB-C18 (1.8 µm, 2.1 mm × 100 mm). The mobile phase consisted of solvent A, pure water with 0.1% formic acid, and solvent B, acetonitrile with 0.1% formic acid. Sample measurements were performed with a gradient program with initial conditions of 95% A and 5% B. Within 9 min, a linear gradient to 5% A and 95% B was programmed, and a composition of 5% A and 95% B was kept for 1 min. Subsequently, a composition of 95% A and 5.0% B was adjusted within 1.1 min and kept for 2.9 min. The column oven was set to 40°C. The injection volume was 4 μl. The effluent was alternatively connected to an ESI-triple quadrupole-linear ion trap (QTRAP)-MS (Li et al., 2021).

### ESI-Q TRAP-MS/MS conditions

Linear ion trap (LIT) and triple quadrupole (QQQ) scans were acquired on a triple quadrupole-LIT mass spectrometer (Q TRAP), AB4500 Q TRAP UPLC/MS/MS System, equipped with an ESI Turbo Ion-Spray interface, operating in positive and negative ion modes and controlled by the Analyst 1.6.3 software (AB Sciex). The ESI source operation parameters were ion source, turbo spray; source temperature of 550°C; ion spray voltage (IS) of 5,500 V (positive ion mode)/–4,500 V (negative ion mode); ion source gas I (GSI), gas II (GSII), and curtain gas (CUR) were set at 50, 60, and 25.0 psi, respectively. The collision gas (CAD) was high. Instrument tuning and mass calibration were performed with 10 and 100 μmol/L polypropylene glycol solutions in QQQ and LIT modes, respectively. QQQ scans were acquired as MRM experiments with collision gas (nitrogen) set to medium. DP and CE for individual MRM transitions were performed with further DP and CE optimization. A specific set of MRM transitions were monitored for each period according to the metabolites eluted within this period (Yang et al., 2021).

### Screening of key active ingredients and target prediction

Metabolites having fold change values ≥2 or ≤0.5 and VIP values ≥1 were identified as differential (Yang et al., 2021). The Venn diagram was used to screen overlapping differential metabolites in each comparison group. Next, the upregulated compounds were further screened from these overlapping metabolites. Afterward, the targets of those compounds were obtained by searching the Traditional Chinese Medicine Systems Pharmacology (TCMSP) database and the Swiss Target Prediction database (Song et al., 2021).

### Alzheimer’s disease target collection and potential target prediction

GeneCards database (Stelzer et al., 2016), National Center for Biotechnology Information (NCBI) gene database (Geer et al., 2010), OMIM database (Amberger et al., 2019) (Online Mendelian Inheritance in Man, https://omim.org/), and Drugbank database (https://go.drugbank.com/) were used to search AD encoding genes with “AD” as the keyword as described in the literature (Jarrell et al., 2018). Venny 2.1.0 was used to map the drug targets of PR to disease targets of AD.

### Gene Ontology enrichment and Kyoto Encyclopedia of Genes and Genomes analysis

The disease-related targets obtained from screening were input into the DAVID database (Huang et al., 2009) by entering the list of target gene names and selecting the species as “*Homo sapiens*.” All target genes were named to their official gene symbols. To perform Gene Ontology (GO) enrichment, the threshold was set to the *p* < 0.01 (or equal) and the WeChat online mapping website was used to visualize the analysis results. Furthermore, the core target was imported into the KOBAS3.0 database, and the Kyoto Encyclopedia of Genes and Genomes (KEGG) function was used to perform pathway analysis (Chen et al., 2017). Taking *p* < 0.05 (or equal) was used to identify pathways and the top 20 KEGG pathways that met the conditions were selected.

### Molecular docking

The PubChem database to find the active ingredients and download its 3D structure. The structure of protein receptors was obtained from the protein data bank (PDB) (Berman et al., 2000). The PyMOL software (The PyMOL Molecular Graphics System, Version 2.0) was used to perform dehydration/ligand/receptor analysis, and the AutoDock 4.2.6 software (Pinzi and Rastelli, 2019) was used to perform hydrogenation/charge calculation on proteins. The receptor protein and ligand small molecules were converted into the pdbqt format and AutoDock Vina 1.1.2 (Trott and Olson, 2010) was used to dock the three receptor proteins with three ligands of small molecules.

### *In vitro* acetylcholinesterase inhibitory assay

The inhibitory of AChE activity was assessed using Ellman’s method (Chen et al., 2016). The reaction system included 10 µl of the sample, 30 µl of 0.05 mol/L phosphate-buffered solution (PBS), 20 µl of AChE solution, 60 µl of 3.75 mmol/L ASCh, and 80 µl of 0.25 mg/ml DTNB and was incubated for 60 min at 37°C. The absorbance intensity of the AChE reaction system was quantified at 412 nm (Nagumo et al., 2019).

### Validation of compounds by *in vitro* assays

#### Cell culture and treatment

Rat pheochromocytoma cells (PC12 cells) were maintained in a medium consisting of Dulbecco's modified eagle medium (DMEM) supplemented with 10% fetal bovine serum (FBS, Gibco, Australia) in humidified 5% CO_2_ at 37°C. PC12 cells were plated in 96 well plates at a density of 5,000 cells per well and cultured for 19 h. To determine the neuroprotective effects of agmatine, caftaric acid, isorhamnetin, rutin, cafestol, syringetin-3-O-glucoside, and 3-(3,4-dihydroxyphenyl)acrylaldehyde on PC12 cells (Zhou F. et al., 2020), the cells were pre-treated with these compounds (20 μM). After 24 h of drug intervention, LPS was added to the administration and model groups to reach the final concentration of 200 μM. Culture at 37°C, 5% CO2 saturation humidity for 8 h.

#### CCK-8 assay

Cell viability was examined using the CCK-8 kit. Briefly, after treatment of the cells with LPS, the supernatant was carefully discarded using a versatile centrifuge at 1,000 rpm for 5 min, and the new medium was re-added, 100 µl per well. Thereafter, 10 µl of CCK8 was added to each well and cultured at 37°C for 4 h. The supernatant was removed carefully and 100 µl of dimethyl sulfoxide (DMSO) was added to each well. The absorbance was measured at 450 nm using the Spectra Max M5 microplate reader (Molecular Devices).

#### Measurement of catalase, GSH-Px, superoxide dismutase, and malondialdehyde

After culturing PC12 cells, these were harvested using a cell scraper and centrifuged at 1,200 rpm for 10 min at 4°C after sonication to collect the cell supernatant. The content of MDA and enzyme viability in the cell supernatants were determined according to the instructions provided in MDA, SOD, CAT, and GSH-Px kits.

#### Measurement of IL-1 β, IL-6, TNF-ɑ, and nitric oxide

The levels of IL-1β, IL-6, TNF-ɑ, and NO in PC12 cell supernatants were determined using the IL-1β, IL-6, TNF-ɑ, and NO ELISA kits, respectively, according to the manufacturer’s protocols.

### Validation of key active ingredients by *in vivo* assays

#### Animals and experimental design

The APP/PS1 transgenic mice and wild-type (WT) littermate mice (males, 4-month-old) weighing about 25 g were used. Each cage contained three to four mice. The mice were fed alternately day and night, and the temperature was kept in the range of 22–25°C. This experiment was approved by the Experimental Animal Ethics Committee of Chengdu University of TCM (SYXK2020-124), in line with the guiding principles of the China Ethics Committee. All mice were separated into five groups: WT control (*n* = 8), AD control (*n* = 8), a3-treated AD (*n* = 8), a4-treated AD (*n* = 8), and a5-treated AD (*n* = 8). The a3-treated, a4-treated, and a5-treated groups were administered a daily dose of 40 mg/kg for 8 weeks via the tail vein. The mice in the AD control and WT groups were treated with 0.5% carboxymethylcellulose (CMC). After drug intervention, all mice were sacrificed by excessive anesthesia with isoflurane. The brain was quickly removed and cut from the middle sagittal slit; the left hemisphere was fixed with 4% paraformaldehyde for frozen sections with Congo red. The other side was used for determining the AChE activity and inflammation detection (Xu et al., 2014). The methods used are the same as those used in the *in vitro* assays.

### Statistical methods

All data are presented as mean ± standard error of the mean (SEM). Statistical analysis was performed using the GraphPad Prism version 8.0 software and the significance of each group was verified using a one-way analysis of variance (ANOVA). A *p*-value < 0.05 was considered significant, whereas a *p*-value < 0.01 represented extreme statistical significance.

## Results

### Metabolic profiling

**FIGURE 2:**
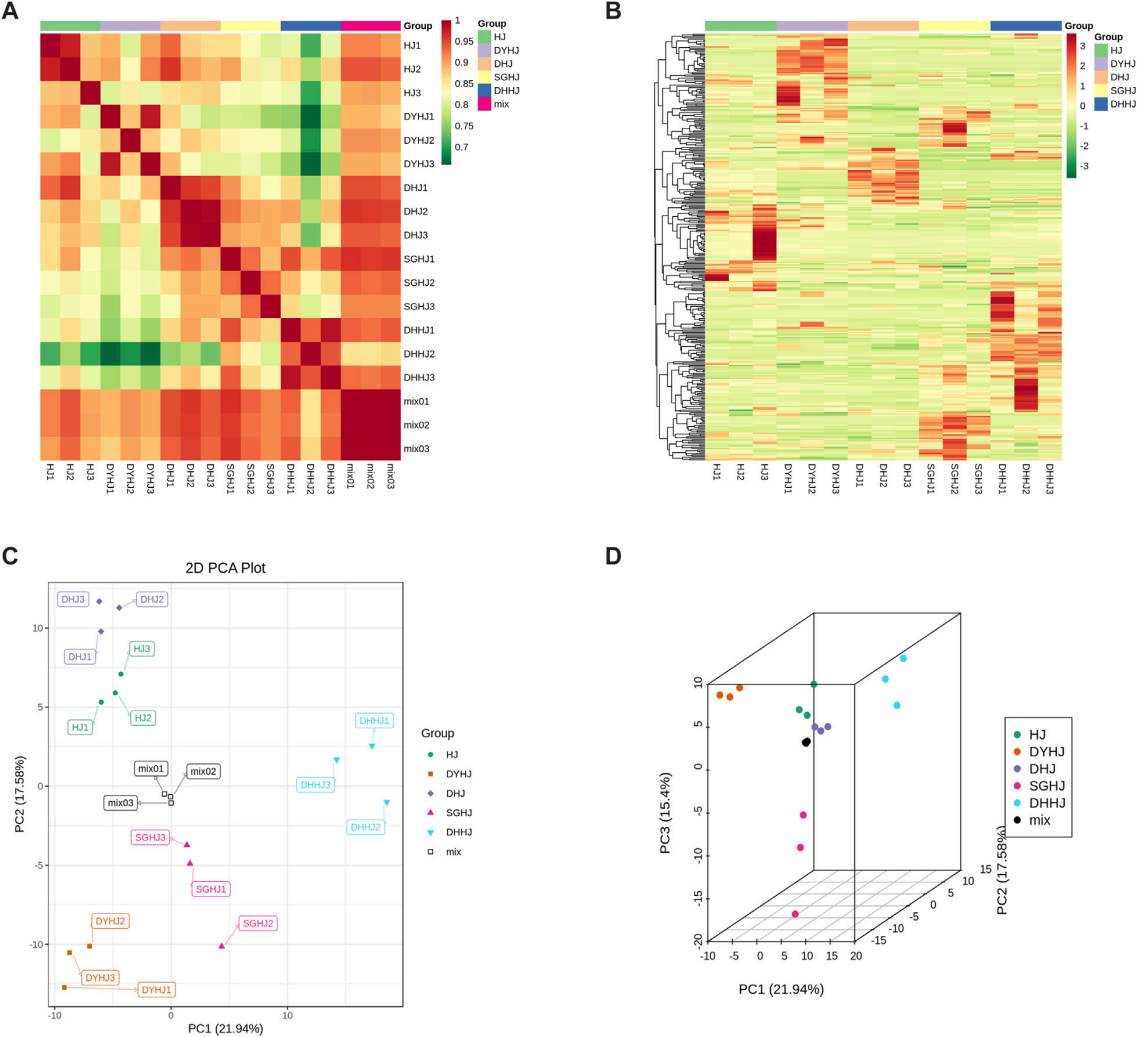
**(A)** Correlation of all the samples. The correlation analysis between the samples was used to estimate the biological duplication among samples within a group. The closer R^2^ is to 1, the stronger the correlation between the two repeated samples. **(B)** Clustering heat-map of metabolites of all the samples. The upregulated and downregulated metabolites were expressed with different shade colors of red and green, respectively. With the increase in the abundance value, the color of the bar changed from green to red. When the abundance value was 0, the color of the bar was white, as shown in the bar at the upper right. **(C)** Differential metabolites analysis on the basis of principal component analysis (PCA). **(D)** PCA 3D plots.

In the present study, the metabolites of PR from SGHJ, HJ, DHJ, DHHJ, and DYHJ were investigated using UPLC-ESI-MS/MS. A total of 335 metabolites were detected and identified in these samples, including 51 alkaloids, 105 flavonoids, 25 lignans and coumarins, 91 phenolic acids, 5 quinones, 23 steroids, 3 tannins, 11 terpenoids, and 21 other compounds (Supplementary Table S1). Figure 2A shows that the correlation coefficient *R* ^2^ within the group was greater than 0.9 in all cases, indicating good repeatability between the samples. The heatmap (Figure 2B) shows that the number of upregulated metabolites was the lowest in SGHJ, when compared with HJ, DHJ, DHJJ, and DYHJ. Although the samples in SGHJ, HJ, DHJ, DHJJ, and DYHJ were grouped, the content of the metabolites considerably differed within each set. This finding was demonstrated by clustering analysis of five samples, which revealed that they could be distinguished from each other.

### Analyses of differential metabolites

Unsupervised principal component analysis (PCA) was performed using the statistics function prcomp within R (www.r-project.org). Unit variance scaling was applied to the data before performing unsupervised PCA. This approach has been widely used for quality control of herbal medicines (Giuliani, 2017). In this study, PCA was performed to provide additional insights into the chemical differences between SGHJ versus each of the four other groups. As shown in Figures 2C,D, HJ, DHJ, DHJJ, and DYHJ were separated from SGHJ, and the QC samples accumulated compactly, indicating the reproducibility and reliability of the experiment. Similarly, orthogonal partial least squares discriminant analysis (OPLS-DA) maximizes the variations between groups and is commonly used to screen differential metabolites (Triba et al., 2015). The logFC, *p*-value, and VIP values of each comparison group are shown in Supplementary Tables S2–S5. The OPLS-DA model compared the metabolite content of samples in pairs to evaluate the difference between SGHJ and HJ (R2Y = 1, Q2 = 0.996; Supplementary Figure S1A), between SGHJ and DHJ (R2Y = 1, Q2 = 0.996; Supplementary Figure S1B), between SGHJ and DHHJ (R2Y = 1, Q2 = 0.998; Supplementary Figure S1C), and between SGHJ and DYHJ (R2Y = 1, Q2 = 0.998; Supplementary Figure S1D). Considering that Q2 exceeded 0.9, the OPLS-DA model was found to be stable and reliable, suggesting that it can be used to identify differential metabolites.

**FIGURE 3:**
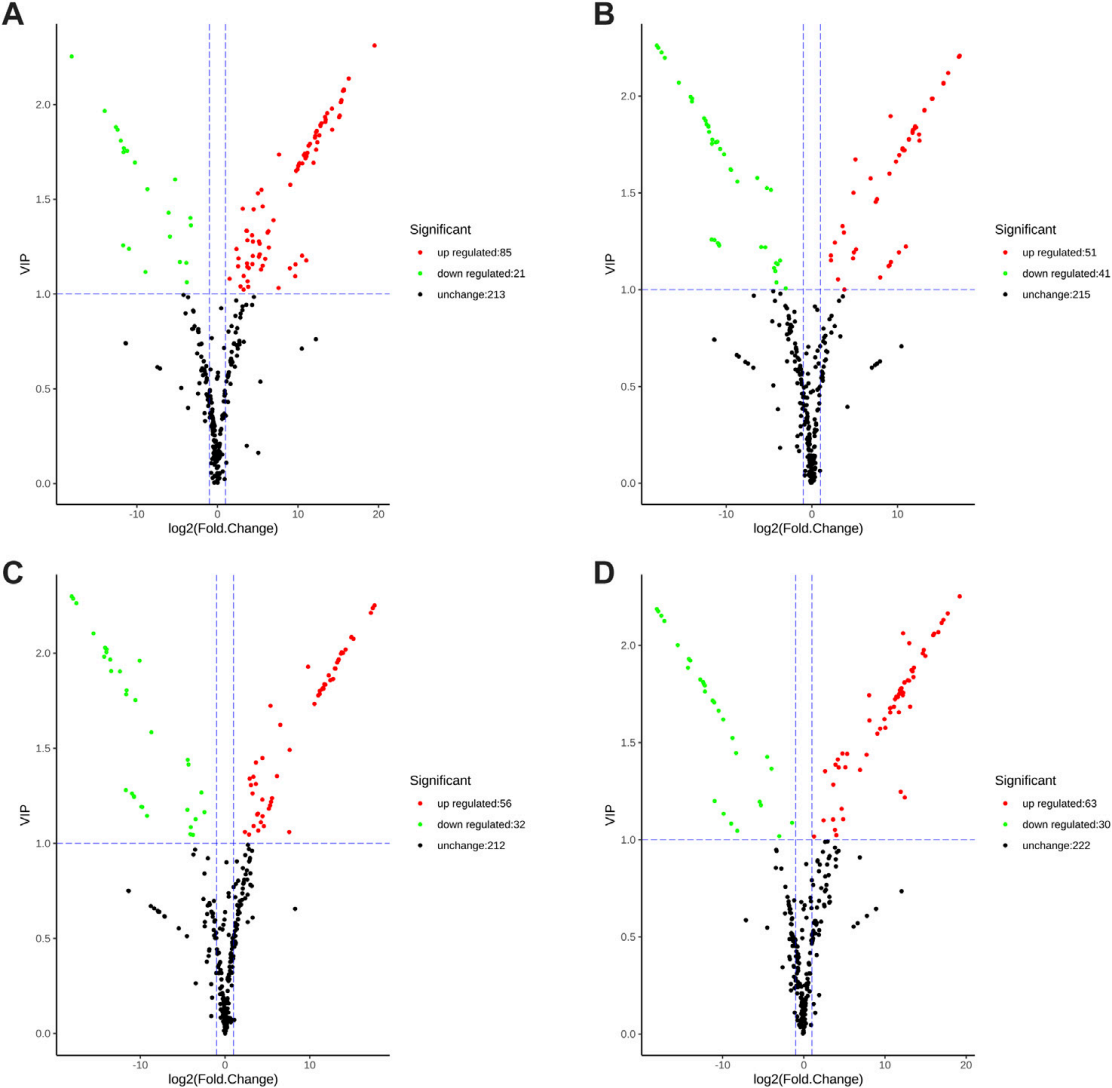
Volcanic plots of the differential metabolites in the comparison of SGHJ versus HJ **(A)**, SGHJ versus DHJ **(B)**, SGHJ versus DYHJ **(C)**, and SGHJ versus DHHJ **(D)**, respectively. Red dots mean upregulated, green dots mean downregulated, and black dots mean no change. SGHJ: *Polygonatum alternicirrhosum* Hand.-Mzt. HJ: *Polygonatum sibiricum* Red. DHJ: *Polygonatum kingianum* Coll.et Hemsl. DHHJ: *Polygonatum cyrtonema* Hua.

Differential metabolites were screened using the fold change and variable importance in project (VIP) values of the OPLS-DA mode. Specifically, metabolites having fold change values ≥2 or ≤0.5 and VIP values ≥1 were identified as differential metabolites. There are 106 significantly differential metabolites between SGHJ and HJ (Figure 3A), including 85 upregulated metabolites and 21 downregulated metabolites. In the comparison group between SGHJ and DHJ, 51 and 41 metabolites were upregulated and downregulated, respectively (Figure 3B). We identified 88 significantly differential metabolites between SGHJ and DHHJ (Figure 3C), including 56 upregulated metabolites and 32 downregulated metabolites. In the comparison group between SGHJ and DYHJ, 63 and 30 metabolites were upregulated and downregulated, respectively (Figure 3D). Notably, compared with the control group SGHJ, more upregulated differential metabolites of all medicinal varieties were identified than downregulated. The results showed that these medicinal varieties were enriched with secondary metabolites, which may be one of the reasons for the difference in the quality between them.

### Screening of key active ingredients of *Polygonati rhizoma*

**FIGURE 4:**
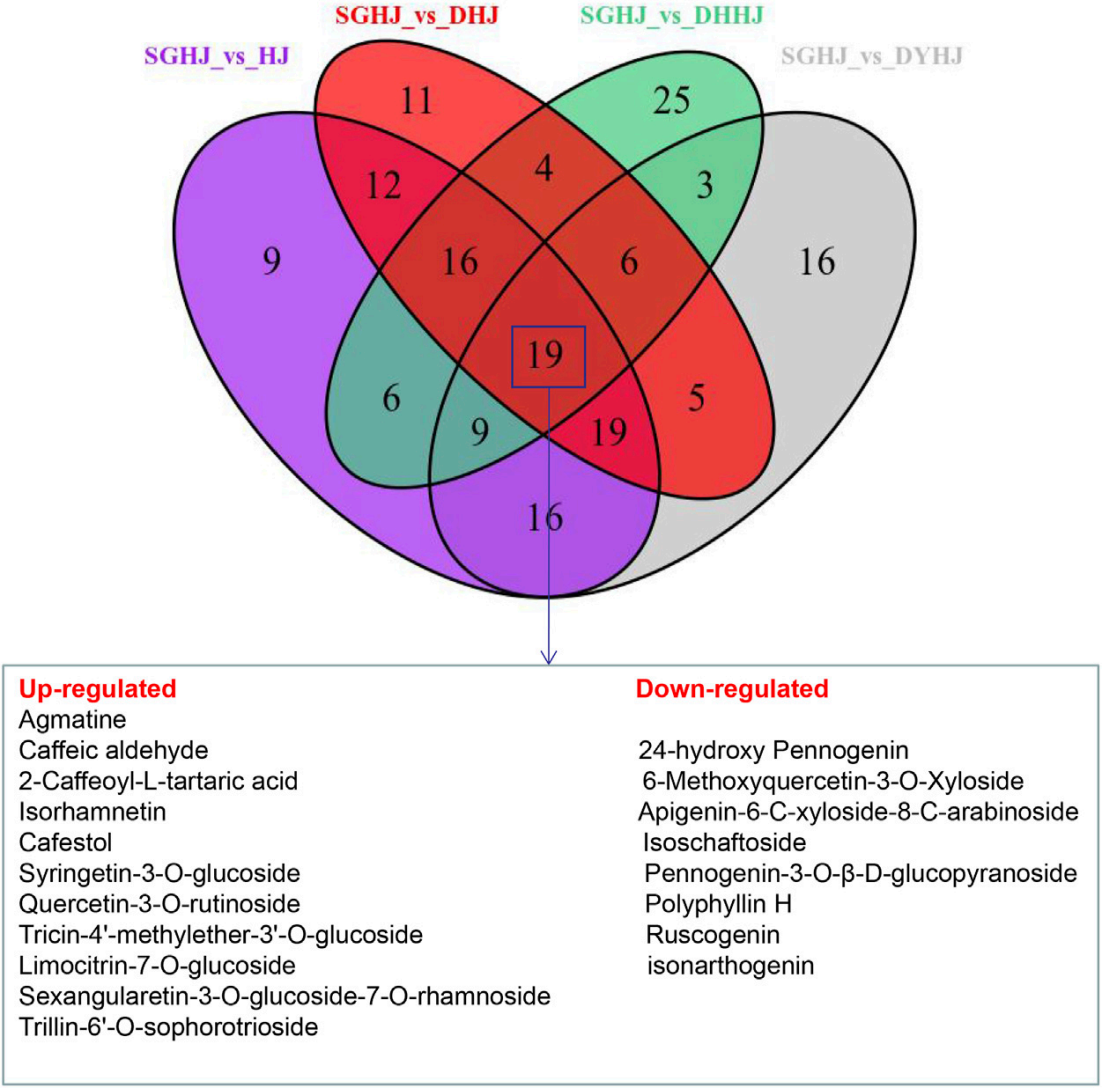
Venn diagrams of the number of different metabolites in the comparison of SGHJ versus HJ, SGHJ versus DHJ, SGHJ versus DYHJ, and SGHJ versus DHHJ, respectively. SGHJ: *Polygonatum alternicirrhosum* Hand.-Mzt. HJ: *Polygonatum sibiricum* Red. DHJ: *Polygonatum kingianum* Coll.et Hemsl. DHHJ: *Polygonatum cyrtonema* Hua.

**FIGURE 5:**
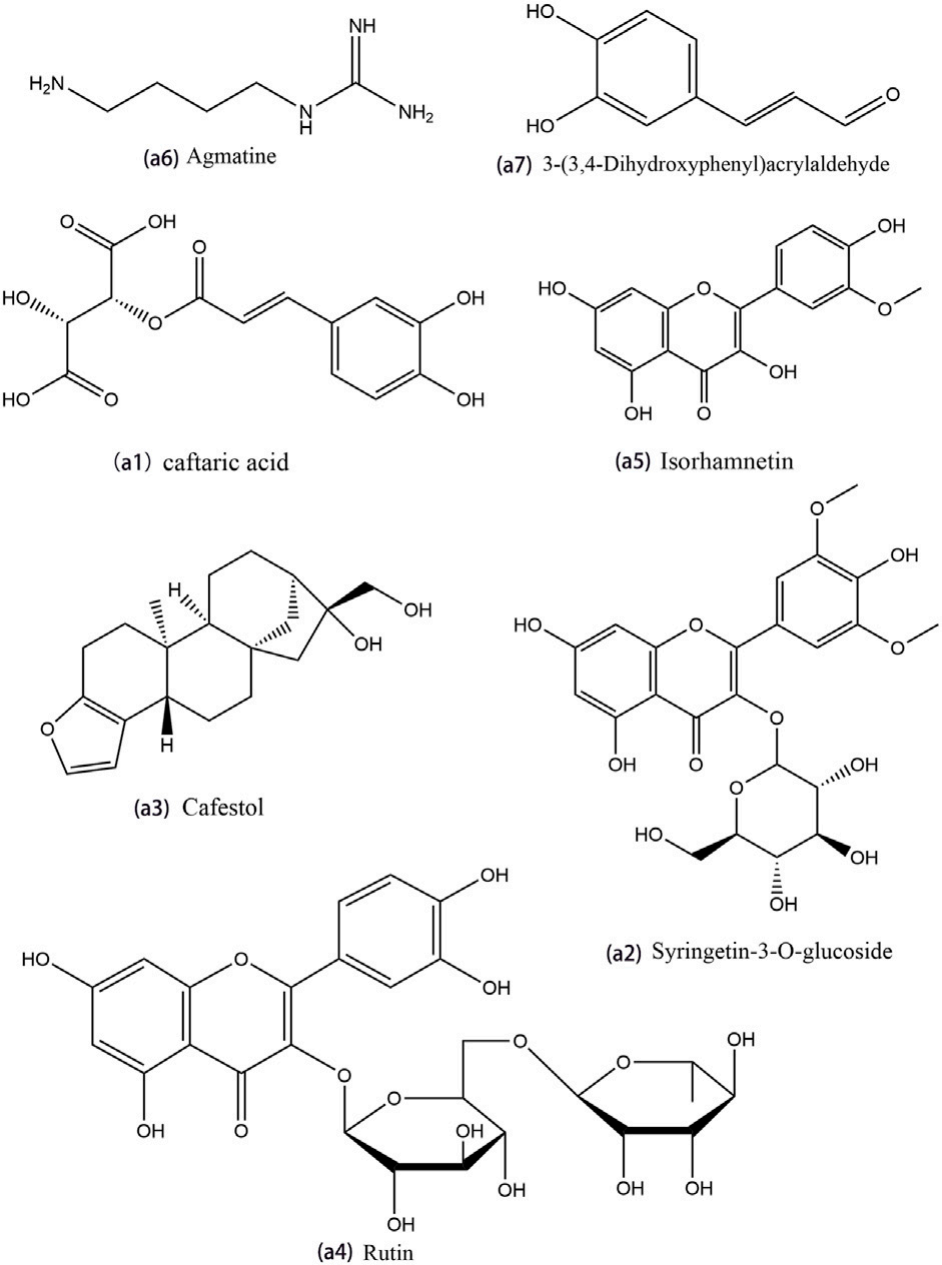
The structure of main compounds in *Polygonati rhzoma* (Huangjing).

The constructed Venn diagram (Figure 4) revealed 19 overlapping differential metabolites from all comparison groups (Table 1). Among them, 11 compounds belonged to the upregulated overlapping differential metabolites and 8 compounds belonged to the downregulated overlapping differential metabolites. Among the upregulated metabolites, agmatine, caftaric acid, isorhamnetin, rutin, cafestol, syringetin-3-O-glucoside, and 3-(3,4-dihydroxyphenyl)acrylaldehyde were identified as key active ingredients because of their significant pharmacological activities (Figure 5).

**TABLE 1:** Overlapping differential metabolites in all the comparison groups.

No.	Q1 (Da)	Q3 (Da)	Molecular weight (Da)	Ionization model	Compounds	VIP	Fold_change	Class	Types
1	131	114	130	[M + H]+	Agmatine	1.45	8.83	Phenolamine	Up
2	165	952	164	[M + H]+	Caffeic aldehyde	1.15	5.92	Phenolic acids	Up
3	311	179	312	[M-H]-	2-Caffeoyl-l-tartaric acid	1.69	1.17E+03	Phenolic acids	Up
4	315	151	316	[M-H]-	Isorhamnetin	2.01	4.07E+04	Flavonols	Up
5	317	299	316	[M + H]+	Cafestol	1.58	5.29E+02	Ditepenoids	Up
6	509	347	508	[M + H]+	Syringetin-3-O-glucoside	1.85	4.85E+03	Flavonoid	Up
7	609	301	610	[M-H]-	Quercetin-3-O-rutinoside	1.18	2.09E+03	Flavonols	Up
8	447	429	446	[M + H]+	24-hydroxy Pennogenin	1.60	2.61E-02	Steroidal saponins	Down
9	465	333	464	[M + H]+	6-Methoxyquercetin-3-O-Xyloside	1.24	4.94E-04	Flavonols	Down
10	479	317	478	[M + H]+	Tricin-4′-methylether-3′-O-glucoside	1.93	3.53E+04	Flavonols	Up
11	509	347	508	[M + H]+	Limocitrin-7-O-glucoside	1.86	5.16E+03	Flavonoid	Up
12	535	517	534	[M + H]+	Apigenin-6-C-xyloside-8-C-arabinoside	1.17	3.94E-02	Flavonoid carbonoside	Down
13	565	409	564	[M + H]+	Isoschaftoside	1.06	7.16E-02	Flavonoid carbonoside	Down
14	593	413	592	[M + H]+	Pennogenin-3-O-β-d-glucopyranoside	1.43	1.50E-02	Steroidal saponins	Down
15	625	317	624	[M + H]+	Sexangularetin-3-O-glucoside-7-O-rhamnoside	1.76	4.87E+03	Flavonols	Up
16	871	413	870	[M + H]+	Polyphyllin H	1.30	1.68E-02	Steroidal saponins	Down
17	927	765	926	[M + H]+	Ruscogenin	1.87	1.85E-04	Steroidal saponins	Down
18	1,040	983	104	[M-H]-	Isonarthogenin	2.25	3.56E-06	Steroidal saponins	Down
19	1060	577	106	[M + H]+	Trillin-6′-O-sophorotrioside	2.14	8.08E+04	Triterpene saponin	Up

Note: Q1 means molecular ion; Q3 means characteristic ion; VIP means variable importance in the projection; Da means dalton.

### Target network analysis of *Polygonati rhizoma*

**FIGURE 6:**
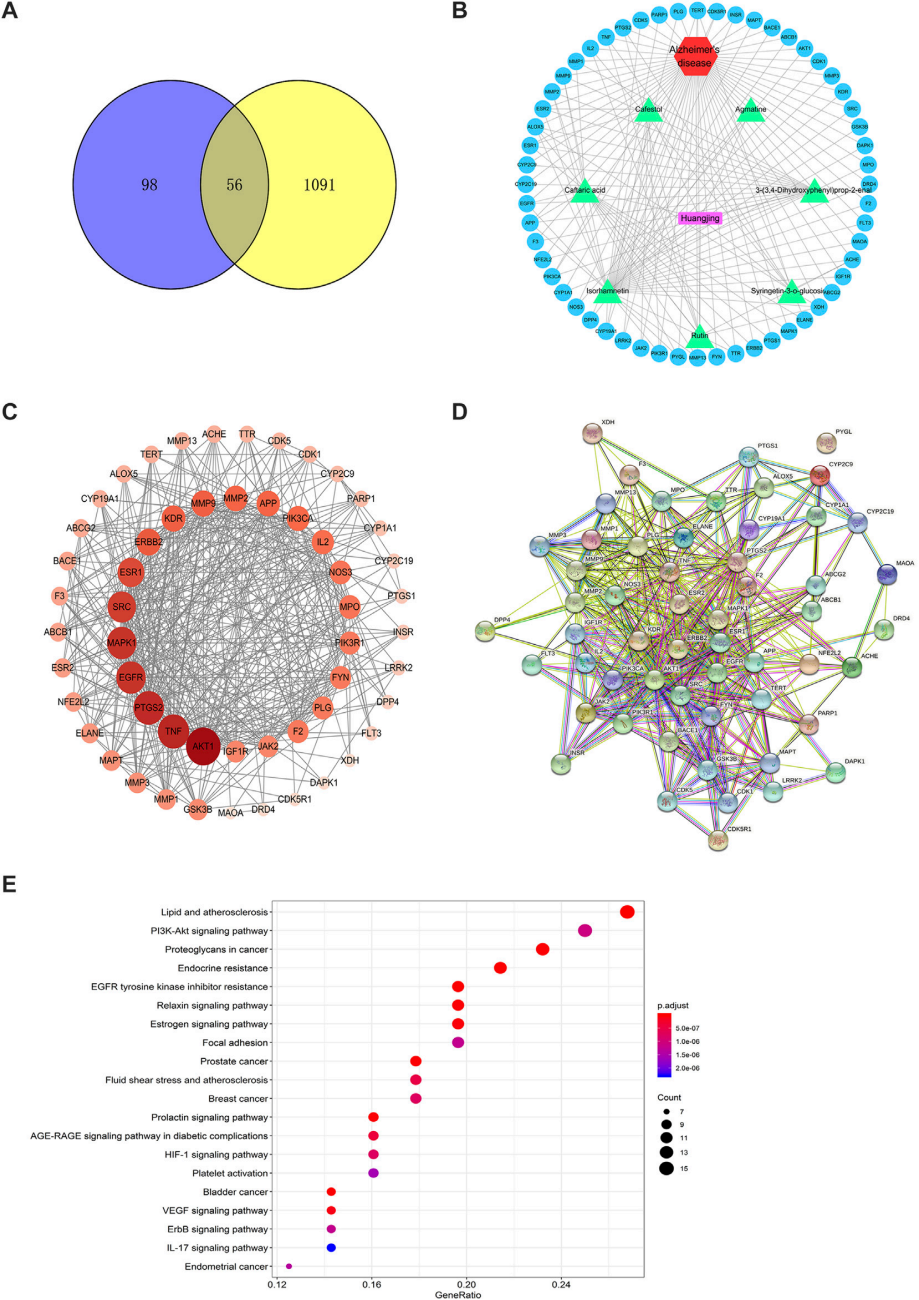
The target network of *Polygonati rhzoma* (Huangjing). **(A)** Components-targets network; **(B)** the Venn Diagram between constituents-related targets and Alzheimer’s Disease-related targets; **(C)** The network of 7 constituents and overlapped Alzheimer’s Disease-related targets; **(D)** The PPI network; **(E)** KEGG analysis of *Polygonati rhzoma*. The size and color were correlated to the degrees of targets in network: the big size and deep color with purple means a high degree of this target.

**FIGURE 7:**
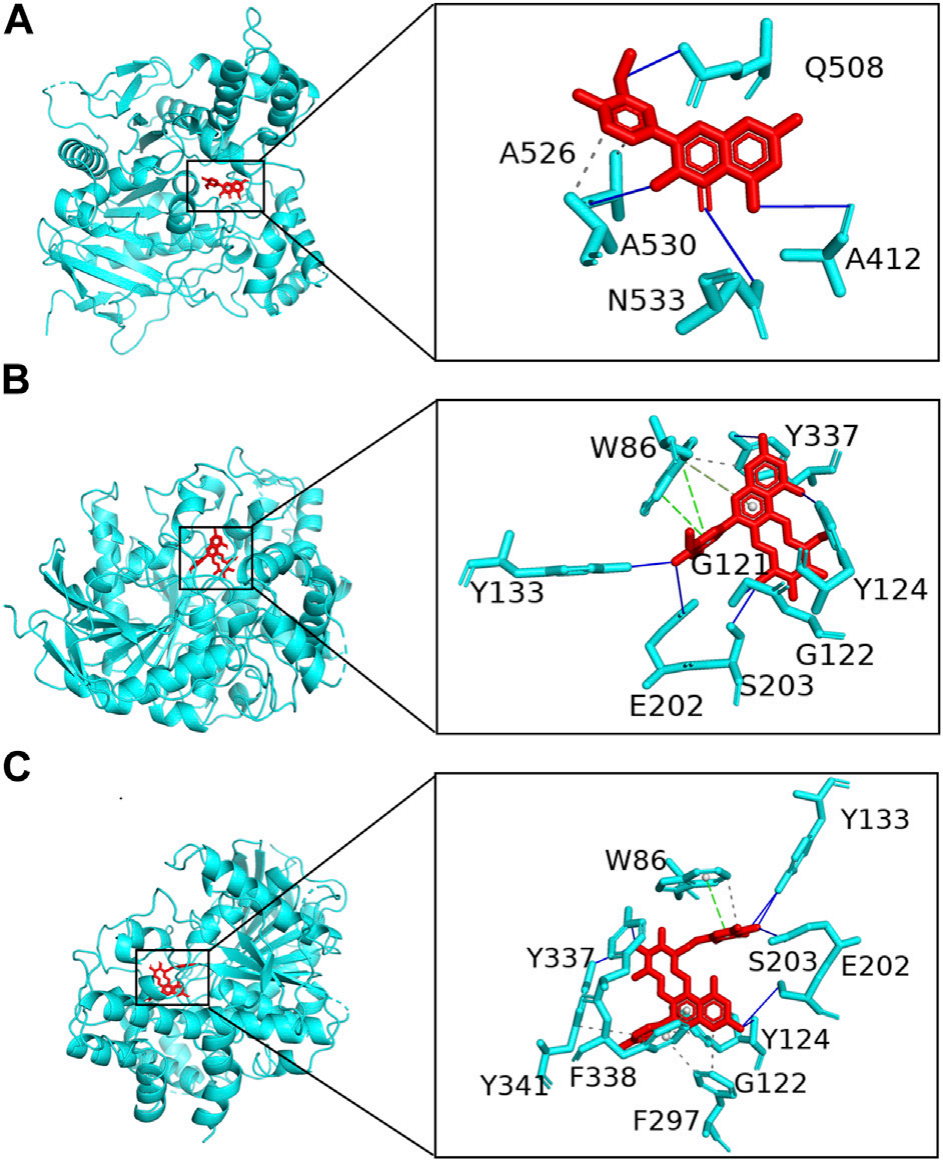
Molecular docking of *Polygonati rhzoma* compounds isorhamnetin **(A)**, rutin **(B)**, and syringetin-3-O-glucoside **(C)** with a target of AChE.

A total of 154 targets of seven key active ingredients (Figure 6A) were obtained using the TCMSP database and Swiss Target Prediction. Based on these data, an ingredient–target network was constructed (Figure 6B). In addition, 1,147 targets of AD were obtained from DisGeNET with a GDA score of more than 0.1. Among these, 56 overlapped targets were found between seven ingredient-related targets and those of AD. Further analysis indicated that all screened key active ingredients targeted AD-related targets (Figure 6C). These genes were used for establishing the PPI network. Among these, ATK1, TNF, EGFR, MAPK1, and AChE were the primary targets (Figure 6D). Interestingly, only rutin, isorhamnetin, and syringetin-3-O-glucoside targeted AChE targets. Moreover, we further verified the above three active ingredients using molecular docking, which revealed that all the three active ingredients had a docking score of less than –5.0 with the target of AChE, indicating that they have strong binding activity. The binding modes between the ingredients and the target of AChE are shown in Figure 7. The molecular docking was performed with rutin, isorhamnetin, and syringetin-3-O-glucoside with AChE target proteins. The results display that the binding pockets of AChE receptor proteins have many amino acid residues that form various bonds with rutin, isorhamnetin, and syringetin-3-O-glucoside, such as A412, G122, Y337, G121, A530, A526, S203, Y133, Y341, F338, F297, and S203.

To further confirm the relationship between targets and the pathways, we analyzed target–pathway interactions using the data extracted from the DAVID database, and the top 20 pathways were screened using the KEGG analysis with BH-corrected *p*-values less than 0.05. The results showed the PI3K–Akt signaling pathway and TNF signal pathway as the primary pathways for these constituents to treat AD (Figure 6E).

### Inhibitory activity of key active ingredients in *Polygonati rhizoma* on acetylcholinesterase

**FIGURE 8:**
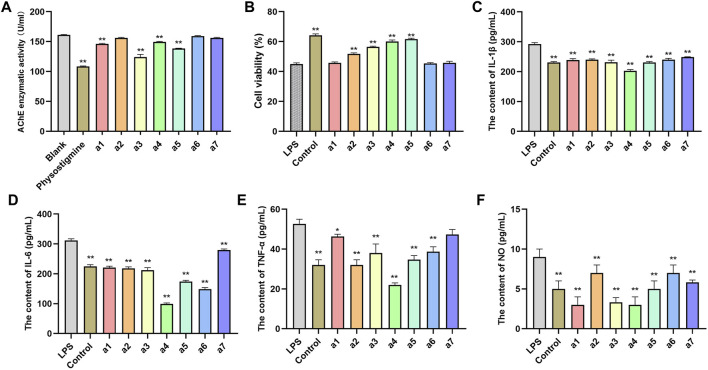
*In vitro* validation of 7 compounds. **(A)** Inhibitory effects of 7 compounds on AChE in PC12 cells. **(B)** Effects of 7 compounds on the viability of PC12 cells. Effect of 7 compounds on the production of IL-1β **(C)**, IL-6 **(D)**, TNF-ɑ **(E)**, and NO **(F)** in LPS-induced PC12 cells. The results represent the mean ± SEM (*n* = 3), vs. LPS *p < 0.05, **p < 0.01.

AChE is one of the major targets of constituents of PR in the treatment of AD. Moreover, it is regarded as one of the drug targets for the treatment of AD (Chen et al., 2016). Hence, seven key active ingredients were further evaluated regarding the inhibitory activity of AChE using *in vitro* assays. At a concentration of 20 μM, the reference compound physostigmine and caftaric acid, cafestol, rutin, and isorhamnetin displayed significant AChE inhibitory activity (*p* < 0.01), compared with that of the blank group. Agmatine, syringetin-3-O-glucoside, and 3-(3,4-dihydroxyphenyl)acrylaldehyde had weak inhibitory activity on AChE (Figure 8A).

### Effects of key active ingredients on the viability of PC12 cells induced by lipopolysaccharide

The lipopolysaccharide (LPS)-induced oxidative stress injury model of PC12 cells was constructed to establish the cell model of AD *in vitro*. PC12 cells cultured *in vitro* were randomly divided into a blank control group, LPS treatment group (model group), and an administration group. As shown in Figure 8B, the cell viability of PC12 cells in the model group after LPS induction was significantly lower than that in the blank group (*p* < 0.01), indicating that LPS successfully induced PC12 cell damage. Compared with the model group, syringetin-3-O-glucoside, cafestol, rutin, and isorhamnetin significantly improved the viability of PC12 cells (*p* < 0.05) and protected PC12 cells from LPS-induced injury. Agmatine, caftaric acid, and 3-(3,4-dihydroxyphenyl)acrylaldehyde exerted no protective effect on LPS-induced PC12 cell injury (Figure 8B).

### Anti-inflammation effects of key active ingredients

The pathophysiology of AD is closely related to inflammation of the central nervous system (Ozben and Ozben, 2019). The anti-inflammatory effect of seven key active ingredients was evaluated using LPS-induced inflammation in PC12 cells. Compared with the control group, the levels of IL-1β, IL-6, TNF-ɑ, and NO in PC12 cells in the model group were significantly increased (*p* < 0.01). Compared with the model group, the levels of IL-1β, IL-6, TNF-ɑ, and NO in the culture medium of PC12 cells in the administration group were significantly decreased (*p* < 0.05, *p* < 0.01), suggesting that all compounds inhibited the inflammatory response in PC12 cells (Figures 8C–F).

### Effects of key active ingredients on the level of malondialdehyde and activities of superoxide dismutase, catalase, and GSH-Px in LPS-induced PC12 cells

Compared with the blank group, the MDA content of lipid peroxidation final product in the model group was significantly increased (*p* < 0.01), and activities of antioxidant enzymes SOD, CAT, and GSH-Px were significantly decreased (*p* < 0.01), indicating that LPS successfully induced oxidative stress in PC12 cells. Compared with the model group, caftaric acid, isorhamnetin, rutin, cafestol, and syringetin-3-O-glucoside significantly reduced the content of MDA and significantly increased the activities of SOD, CAT, and GSH-Px (*p* < 0.05). The results showed that caftaric acid, isorhamnetin, rutin, cafestol, and syringetin-3-O-glucoside significantly reduced oxidative stress injury in the LPS-induced AD cell model. However, agmatine and 3-(3,4-dihydroxyphenyl)acrylaldehyde could not significantly reduce the content of MDA, showing that they did not significantly reduce the oxidative stress injury in the LPS-induced AD cell model (Table 2).

**TABLE 2:** Effect of 7 key active ingredients on the changes of MDA and antioxidant enzyme activity in PC12 cells induced by LPS.

Group	MDA (μmol/L)	SOD (U/ml)	CAT (U/ml)	GSH-Px (U/ml)
Blank	1.11 ± 0.34	7.11 ± 0.67	103.26 ± 2.00	419.42 ± 5.54
Model	9.21 ± 0.67^##^	0.16 ± 0.34^##^	21.06 ± 0.67^##^	88.81 ± 3.20^##^
a1	5.35 ± 0.24**	1.94 ± 0.61**	89.05 ± 0.47**	229.21 ± 5.54**
a2	5.57 ± 0.46**	2.18 ± 0.74**	93.50 ± 0.36**	290.88 ± 6.33**
a3	5.38 ± 1.15**	2.18 ± 0.56**	92.83 ± 1.09**	223.67 ± 11.42**
a4	6.85 ± 0.46*	2.44 ± 0.46**	92.00 ± 0.54**	223.67 ± 11.42**
a5	3.85 ± 0.01**	2.45 ± 0.39**	89.21 ± 0.59**	108.07 ± 11.09**
a6	7.82 ± 1.19	2.42 ± 1.19**	87.25 ± 0.20**	319.79 ± 30.55**
a7	9.01 ± 1.22	2.61 ± 0.22**	85.98 ± 0.84**	143.16 ± 8.47**

Note: The results represent the mean ± SEM (*n* = 3), vs. Normal blank ^##^ *p* < 0.01; vs. Model *p < 0.05, **p < 0.01.

### Effects of key active ingredients on Aβ deposition in the hippocampus of APP/PS1 Alzheimer’s disease model mice

**FIGURE 9:**
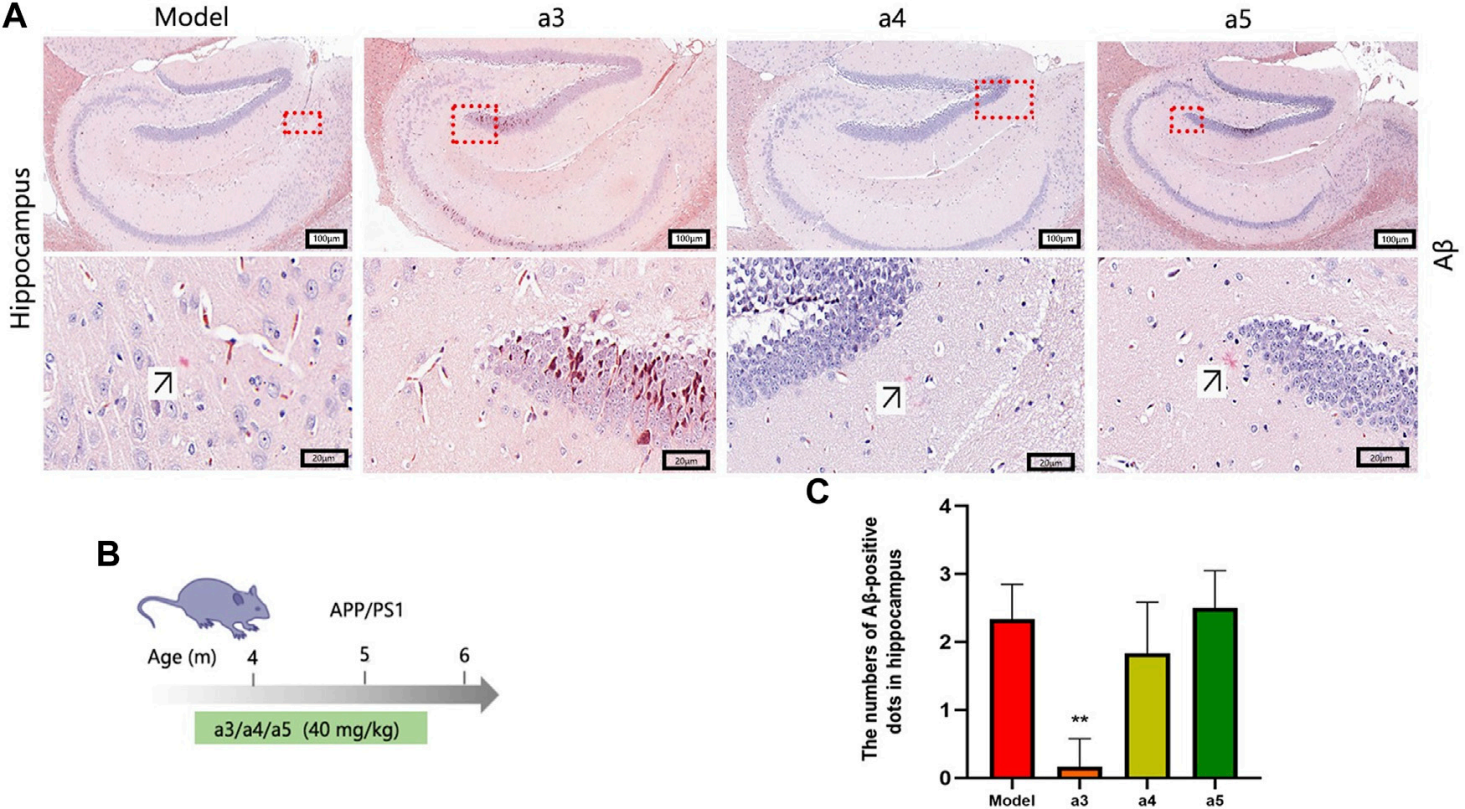
Levels of Aβ deposition in the hippocampus. **(A)** Congo red staining of the hippocampus. **(B)** Schematic of the protocol for dose and time in APP/PS1 mice. **(C)** Aβ-positive dots in the hippocampus.

The above *in vitro* validation assays showed that cafestol, isorhamnetin, and rutin have AChE inhibitory, anti-inflammatory, and neuroprotective effects. Therefore, they were selected for further *in vivo* experiments to assess their therapeutic potential against AD. In this part, APP/PS1 (Series no: c000111) AD mice were used as the model. APP/PS1 is a double transgenic mouse expressing a chimeric murine/human amyloid precursor protein (Mo/Huap695Swe) and mutated human presenilin 1 (PS1-DE9), both targeting the neurons in the central nervous system (Heneka et al., 2013). Both mutations have been linked to early-onset AD. The transgenic mice developed Aβ deposits in their brains when they were 6 months old. To investigate the effects of the three key active ingredients on Aβ deposition in the hippocampal area of APP/PS1 mice at the early stage of disease, we selected 4-month-old mice as experimental materials and administered them these ingredients continuously for 2 months (Figure 9B). Congo red staining was used to detect the deposition of Aβ in the hippocampus of each group. Aβ-positive spots were found in the hippocampus of AD model mice (Figure 9A). Compared with the AD model mice, the number of Aβ-positive spots in the hippocampus of the cafestol treatment group was significantly reduced (*p* < 0.01). However, the number of Aβ-positive spots in the hippocampus of isorhamnetin and rutin-treated mice did not decrease significantly (*p* > 0.05) (Figure 9C).

### Effects of key active ingredients on oxidative stress in the brain of Alzheimer’s disease model mice

Compared with the blank group, the MDA content of lipid peroxidation final product in the model group was significantly increased (*p* < 0.01), and the activities of antioxidant enzymes SOD, CAT, and GSH-Px were significantly decreased (*p* < 0.01), suggesting that the level of oxidative stress in the model group was higher than that in the blank group. Compared with the model group, cafestol, rutin, and isorhamnetin significantly reduce the content of MDA and significantly increase the activities of SOD, CAT, and GSH-Px (*p* < 0.05, *p* < 0.01). The results showed that cafestol, rutin, and isorhamnetin significantly inhibited the level of oxidative stress in the brain of AD model mice (Table 3).

**TABLE 3:** Effect of 3 key active ingredients on the changes of MDA and antioxidant enzyme activity in hippocampus of App/PS1 mice.

Group	MDA (umol/g)	SOD(U/mg)	CAT(U/mg)	GSH-Px (U/mg)
Blank	10.00 ± 2.10	52.49 ± 3.75	7.11 ± 0.84	1.20 ± 0.11
Model	21.83 ± 0.12^##^	27.81 ± 5.53^##^	2.61 ± 0.16^##^	0.31 ± 0.04^##^
a3	13.11 ± 4.30*	42.58 ± 1.78*	4.61 ± 0.57**	0.61 ± 0.05*
a4	11.13 ± 3.24**	41.57 ± 1.52*	5.02 ± 0.94**	1.65 ± 0.37**
a5	10.14 ± 2.12**	41.33 ± 4.55*	3.77 ± 0.39^*^	1.09 ± 0.17**

Note: The results represent the mean ± SEM (*n* = 6), vs. Normal blank ^##^ *p* < 0.01; vs. Model *p < 0.05, **p < 0.01.

### Inhibitory activity of key active ingredients on acetylcholinesterase in the brain of Alzheimer’s disease model mice

**FIGURE 10:**
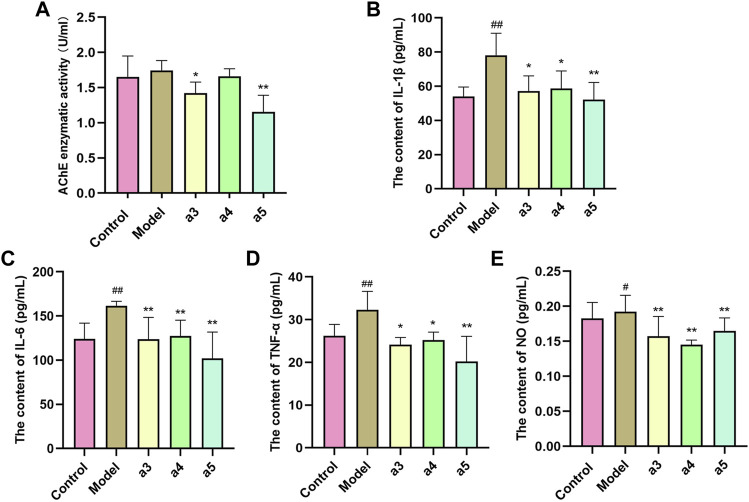
*In vivo* validation of 3 key active ingredients. **(A)** Inhibitory effects of 3 key active ingredients on AChE in the hippocampus of APP/PS1 mice. Effect of 3 key active ingredients on the production of IL-1β **(B)**, IL-6 **(C)**, TNF-ɑ **(D)**, and NO **(E)** in the hippocampus of APP/PS1 mice. The results represent the mean ± SEM (*n* = 6), vs. Normal blank ^##^ *p* < 0.01; vs. Model *p < 0.05, **p < 0.01.

To further verify the inhibitory effect of the three key active ingredients on the activity of AChE in the brain of AD model mice, we measured the activity of AChE in the hippocampus of mice in each comparison group by ELISA. Cafestol and isorhamnetin displayed significant AChE inhibitory activity (*p* < 0.05, *p* < 0.01), compared to that of the model group (Figure 10A).

### Anti-inflammation effects of three key active ingredients in the brain of Alzheimer’s disease model mice

Compared with the control group, the levels of IL-1β, IL-6, TNF-ɑ, and NO in the brain of AD mice in the model group were significantly increased (*p* < 0.05, *p* < 0.01), suggesting inflammation in the brain of the model group mice. Compared with the model group, the levels of IL-1β, IL-6, TNF-ɑ, and NO in the brain of AD mice in the administration group were significantly decreased (*p* < 0.05, *p* < 0.01), suggesting that all the three key active ingredients inhibited the inflammatory response in the brains of AD mice (Figures 10B–E).

## Discussion

Since Alois Alzheimer reported the first case of AD in 1907, there has been no proven cure for the disease (Zhu et al., 2022). Currently, drugs can alleviate the symptoms of AD to a certain extent; however, the therapeutic effect is unsatisfactory, and the side effects are great (Kosel et al., 2020). Faced with complex diseases, an increasing number of researchers are diverting their attention to traditional medicines, such as TCM. TCM is generally a natural product after long-term clinical practice, with good safety and low toxicity. In addition, TCM is characterized by the use of multiple compounds, multiple targets, and multiple pathways of action, thus having certain advantages for complex diseases (Fang et al., 2020). Thus, we assumed that the treatment of PR might be ascribed to the integrated effects of multiple compounds, rather than a single constituent.

However, TCM often contains hundreds or even thousands of ingredients, with only a few compounds having medicinal and/or toxic effects (Ji et al., 2021). The numerous other components present in TCM make the screening and analysis of bioactive components extremely difficult, which is a great challenge to the research on TCM. In recent years, with the continuous progress in several high-throughput assembly technologies and the rapid development of computer technology, new strategies have emerged to study the material basis of TCM, such as comparative metabolomics, which is an emerging system biology technology that compares the metabolites of the control and experimental groups to identify the differences in their metabolic profiles (Rashid et al., 2021). Researchers divide herbal medicines into control and experimental groups, and further screen active ingredients based on differential metabolites. In addition, network pharmacology is often associated with comparative metabolic methods. Researchers could understand herbal medicines and diseases better with the network pharmacology method.

In this work, an integrative approach of comparative metabolomics and network pharmacology was used to investigate the material basis and their therapeutic potential in PR for treating AD. According to clinical and pharmacological experimental design methods, edible PR was used as the control group (SGHJ), and medicinal PR (HJ, DHJ, DHHJ, and DYHJ) were used as the experimental group. In total, 335 metabolites were identified, and 19 overlapping differential metabolites were obtained from the comparison groups. Among these differential metabolites, nine compounds were found to be upregulated and seven of them were further identified as the key active ingredients of PR according to their availability and bioactivity. Network pharmacology analysis indicated that all seven key active ingredients targeted AD-related targets. For example, ATK1, TNF, EGFR, MAPK1, and AChE were the primary targets. Different active compounds not only act on different targets to affect the same pathways but also act on the same targets to affect different pathways, indicating that different targets of different compounds exert a synergistic effect, reflecting the characteristics of multi-compounds, multi-targets, and multi-action pathways of TCM.

Among those primary targets, AChE is a key enzyme in biological nerve conduction, which catalyzes the hydrolysis of the neurotransmitter acetylcholine (ACh) to choline and acetic acid. Previous research studies have suggested that inhibiting the enzyme AChE, which breaks down ACh, is a promising strategy for treating patients with AD (Ferreira-Vieira et al., 2016). Although there exist alternative hypotheses, such as the amyloid and tau hypotheses, AChE is a favorable enzyme target for numerous researchers. In the present study, target network analysis of PR showed that only rutin, isorhamnetin, and syringetin-3-O-glucoside targeted AChE targets. Molecular docking analysis of the three key active ingredients confirmed that they possessed strong molecular interactions at the potential ligand-binding site of AChE. To further verify the results of the prediction, all the seven key active ingredients were further evaluated regarding the inhibitory activity of AChE using *in vitro* assays. Our data showed that caftaric acid, cafestol, rutin, and isorhamnetin displayed significant AChE inhibitory activity. However, syringetin-3-O-glucoside had weak inhibitory activity on AChE. Simultaneously, *in vitro* cell validation experiments have demonstrated that cafestol, rutin, and isorhamnetin improved nerve cell vitality, anti-inflammatory, and reduced oxidative stress injury, respectively (Table 4). Hence, we finally selected cafestol, isorhamnetin, and rutin for *in vivo* validation.

**TABLE 4:** *In vivo* and *in vitro* evaluation of potential key active ingredients from Polygonati rhzoma in the treatment of Alzheimer’s disease.

Compounds	Inhibition of AChE *in vitro*	Inhibition of AChE *in vivo*	Attenuating oxidative stress *in vitro*	Attenuating oxidative stress *in vivo*	Anti-inflammation *in vitro*	Anti-inflammation *in vivo*	Neuroprotection *in vitro*	Reducing aβ
a1	√		√		√			
a2			√		√		√	
a3	√	√	√	√	√	√	√	√
a4	√		√	√	√	√	√	
a5	√	√	√	√	√	√	√	
a6					√			
a7					√			

AD is a multifactorial and complex disease. Damage to the cholinergic system and oxidative stress are two important mechanisms leading to AD (Kamat et al., 2016). The primary manifestations of cholinergic injury are the increased activity of AChE and loss of ACh. Oxidative stress is a dynamic state of instability between the levels of peroxides and antioxidants. The increase in the MDA content of lipid peroxides and the decrease in the activities of antioxidant enzymes, such as SOD, CAT, and GSH-Px, are important manifestations of oxidative stress response in AD. In the present work, cafestol, isorhamnetin, and rutin demonstrated antioxidant activity by significantly reducing the MDA levels, and increasing SOD, CAT, and GSH-Px activities in AD mice brains, which is consistent with our *in vitro* results. Furthermore, cafestol and isorhamnetin displayed significant AChE inhibitory activity in the brain of AD model mice (Table 4). This result indicates that cafestol and isorhamnetin reduced cholinergic injury by inhibiting the activity of AChE. Moreover, it increased the activity of antioxidant enzymes such as SOD, CAT, and GSH-Px, and reduced the content of the lipid peroxidation end product MDA, thus alleviating the oxidative stress damage in the brain of AD mice.

During the progression of AD, Aβ plays a critical and primary role in the pathogenesis of AD (Tiwari et al., 2019). The glial cells around Aβ plaques release a wide variety of pro-inflammatory cytokines, such as IL-1β, IL-6, TNF-ɑ, and NO (Li et al., 2017). In this study, we primarily focused on whether the key active ingredients could reduce the number of Aβ-positive spots during the early stage in the brain of AD mice. Our data showed that only cafestol significantly reduced the number of Aβ-positive spots in the hippocampus of AD mice. Furthermore, cafestol, isorhamnetin, and rutin inhibited the inflammatory response in the brain of AD mice. Together, the screened key active ingredients display several beneficial effects in AD transgenic mice by reducing the number of Aβ-positive spots and the levels of inflammatory cytokines, inhibiting AChE activity, and increasing antioxidant levels. Each compound plays a different role in the progression of AD. Xie et al. reported that PR extract may against kinases through multiple pathways, and it could have the potential to be used to inhibit cell growth in combination with kinase inhibitors, which might be applied in treatments against other types of diseases in the future (Xie et al., 2021). Other previous reports also suggested that PR has a great potential in treatment of COVID-19 through multiple components and multiple pathways (Mu et al., 2021). Hence, we conclude that the treatment of AD using PR could be ascribed to the integrated effects of multiple compounds rather than a single constituent, which coincides with the characteristics of TCM and the holistic view of TCM treatment.

In conclusion, our study is the first to elucidate the material basis and synergistic mechanism of the constituents in PR for the potential treatment of AD using comparative metabolomics and network pharmacology. Seven overlapping upregulated differential metabolites were identified as key active ingredients. Among them, cafestol, isorhamnetin, and rutin are potential lead compounds in PR for the treatment of AD, which was verified by virtual validation, *in vitro,* and *in vivo* validation. The study was expected to broaden the options of AD treatment methods and further demonstrate the feasibility to apply comparative metabolomics and network pharmacology to the analysis of TCM.

## Data Availability

The original contributions presented in the study are included in the article/Supplementary Material, further inquiries can be directed to the corresponding authors.
