# Supplementary figures and images for: Integrated comparative metabolomics and network pharmacology approach to uncover the key active ingredients of Polygonati rhizoma and their therapeutic potential for the treatment of Alzheimer’s disease
Source: Front Pharmacol. 2022 Aug 4;13:934947. doi: 10.3389/fphar.2022.934947 (PMC9385993; doi:10.3389/fphar.2022.934947)

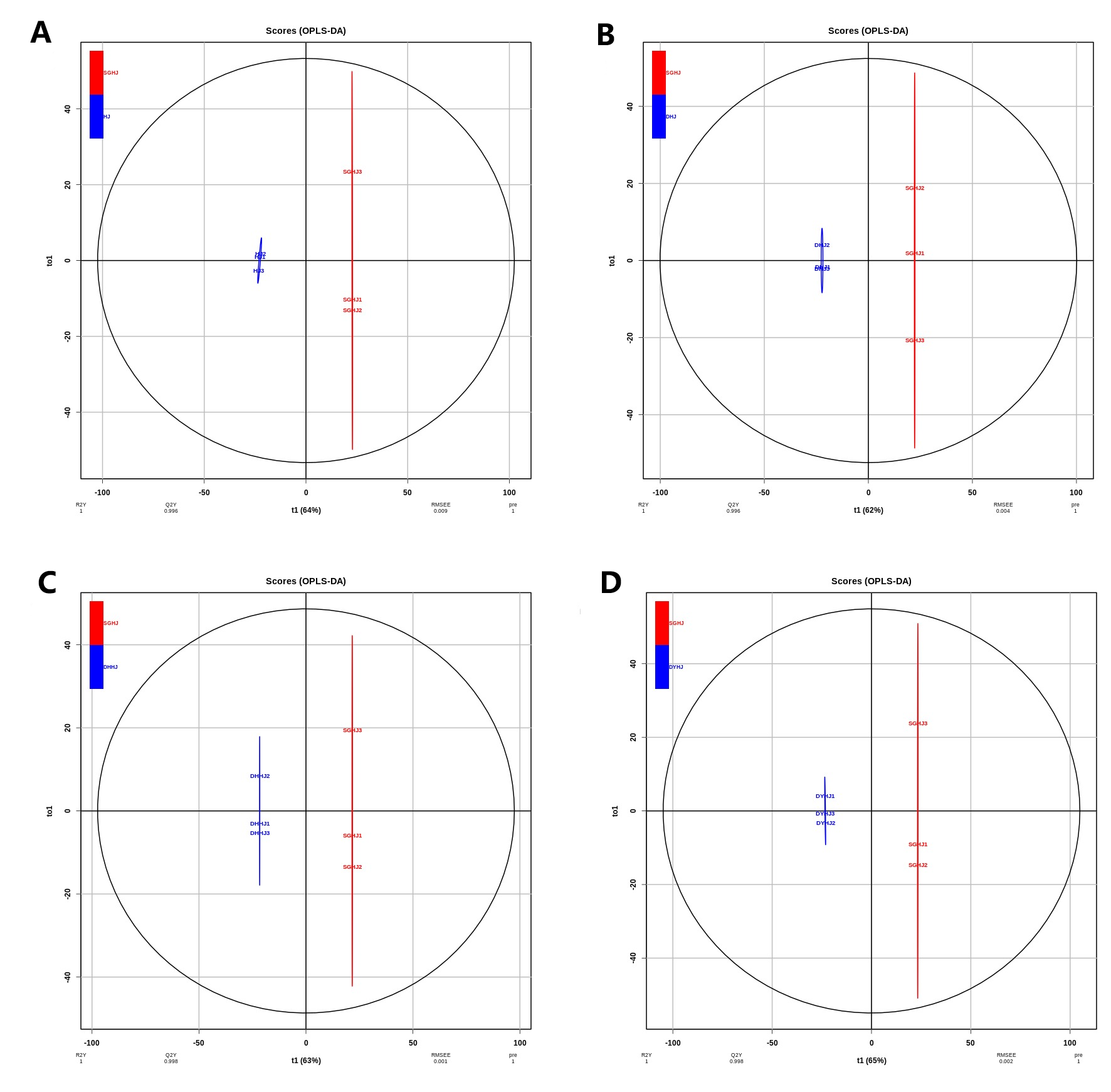

Supplement: Supplementary file 3 [file Image1.TIF]
